# The Effects of Fluctuations in the Nutrient Supply on the Expression of Five Members of the *AGL17* Clade of MADS-Box Genes in Rice

**DOI:** 10.1371/journal.pone.0105597

**Published:** 2014-08-20

**Authors:** Chunyan Yu, Sha Su, Yichun Xu, Yongqin Zhao, An Yan, Linli Huang, Imran Ali, Yinbo Gan

**Affiliations:** Zhejiang Key Lab of Crop Germplasm, Department of Agronomy, College of Agriculture and Biotechnology, Zhejiang University, Hangzhou, China; University of Michigan, United States of America

## Abstract

The *ANR1* MADS-box gene in *Arabidopsis* is a key gene involved in regulating lateral root development in response to the external nitrate supply. There are five *ANR1*-like genes in *Oryza sativa*, *OsMADS23, OsMADS25, OsMADS27, OsMADS57* and *OsMADS61*, all of which belong to the *AGL17* clade. Here we have investigated the responsiveness of these genes to fluctuations in nitrogen (N), phosphorus (P) and sulfur (S) mineral nutrient supply. The MADS-box genes have been shown to have a range of responses to the nutrient supply. The expression of *OsMADS61* was transiently induced by N deprivation but was not affected by re-supply with various N sources. The expression of *OsMADS25* and *OsMADS27* was induced by re-supplying with NO_3_^−^ and NH_4_NO_3_, but downregulated by NH_4_^+^. The expression of *OsMADS57* was significantly downregulated by N starvation and upregulated by 3 h NO_3_^−^ re-supply. *OsMADS23* was the only gene that showed no response to either N starvation nor NO_3_^−^ re-supply. *OsMADS57* was the only gene not regulated by P fluctuation whereas the expression of *OsMADS23, OsMADS25* and *OsMADS27* was downregulated by P starvation and P re-supply. In contrast, all five *ANR1*-related genes were significantly upregulated by S starvation. Our results also indicated that there were interactions among nitrate, sulphate and phosphate transporters in rice.

## Introduction

Nitrogen, phosphorus and sulfur are three major macronutrients essential for plant growth and development [1]. Nitrogen (N) deficiency is a vital factor limiting agricultural quality and productivity. In aerobic soil conditions, nitrate is the major source of nitrogen for many higher plant species [2]–[4]. Nitrate acts not only as a nutrient but also as a signal to regulate gene expression, energy transfer, protein activation, metabolic and physiological activities, and plant growth and development [5]–[8]. Phosphorus (P) is a component of many key biomolecules and participates in various enzymatic reactions and metabolic pathways [9]. Due to low availability of phosphate (Pi) in soil, it acts as the second most important limiting macronutrients for plant growth and development [10]–[12]. Plants can modify their root architecture to improve phosphate acquisition in phosphate deficient soil. Many studies have demonstrated that P deficiency affects root development, including root hair elongation, reduced primary root length and increased number and length of lateral roots in *Arabidopsis thaliana* [13]–[19]. Sulfur (S) is also an essential macronutrients, and its deficiency adversely affects plant growth and development as well as the quality of crops [20], [21].

Plants can modify their root architecture to forage for sources of N that are distributed unevenly in soil [6]. One important kind of foraging response involves increased proliferation of lateral roots within soil patches enriched in certain nutrients, such as NH_4_^+^ and NO_3_^−^ [22], [23]. There are signaling mechanisms in roots to measure the levels of intrinsic and extrinsic nutritional factors so that they can modify their growth and development [24], [25]. In *Arabidopsis*, localized nitrate treatment stimulated a localized increase in lateral root (LR) numbers and elongation. The stimulation of LR elongation was found to be the result of a signaling effect of external NO_3_^−^ ion itself rather than downstream metabolites [26], [27]. An important breakthrough in understanding NO_3_^−^ in stimulating LR growth was the identification of the *ANR1* gene, which is a vital component in regulatory signaling pathway [26]. *ANR1* is a member of the plant MADS-box family of transcription factors, which covers more than 100 members in *Arabidopsis* [28]–[30]. In addition to their roles in regulation of reproductive development, the MADS-box transcription factors are also widely expressed in vegetative tissues [28], [31]. Recent research has demonstrated that at least 50 MADS-box genes are expressed in roots of *Arabidopsis*, with *ANR1* as the only member so far to have a known function in lateral root development in *Arabidopsis* [32], [33]. Previous reports revealed that *ANR1* is a positive regulator of lateral root growth and is not present in the primary root tip [34]. Initial studies using *Arabidopsis* root cultures had demonstrated that *ANR1* gene was NO_3_^–^ inducible [26]. Subsequent results obtained from hydroponically culture experiments illustrated that the expression of *ANR1* was induced by N deprivation and rapidly downregulated when NO_3_^–^ or other N source was re-supplied [29]. *OsMADS23, OsMADS25, OsMADS27, OsMADS57* and *OsMADS61* are five *ANR1*-like homologs in rice and their functions have not been well understood [35], [36]. Recent work from Meng et al. (2013) has suggested that *OsMADS27* could play a key role in the response to cold and salt stress [37]. To gain further insight into the possible regulatory functions of *ANR1*-like genes in roots, we have investigated their root expression patterns in response to N, P, and S fluctuation using quantitative real-time PCR (qPCR).

## Materials and Methods

### Plant Materials

Rice seeds (*Oryza sativa L.* cv. *Nipponbare*) were used for all experiments.

### Hydroponic culture

Rice seeds were surface-sterilized by treatment with 70% ethanol for 1 min and 10% sodium hypochlorite for 20 min, followed by five rinses with sterile distilled water. Seeds were germinated in the dark by placing into the incubator at 28°C for 2 days (d). Uniform seedlings were selected and transferred to black plastic buckets containing 4 L nutrient solution, where the growth conditions were 30/28°C day/night temperature with a 14/10 h light/dark at a relative humidity of 65–70%. The complete nutrient solution contained: 1.44 mM NH_4_NO_3_, 0.32 mM NaH_2_PO_4_, 0.5 mM K_2_SO_4_, 1 mM CaCl_2_·2H_2_O, 1.6 mM MgSO_4_·7H_2_O, 50 µM Fe-EDTA, 15 µM H_3_BO_3_, 9 µM MnCl_2_·4H_2_O, 0.12 µM CuSO_4_·5H_2_O, 0.12 µM ZnSO_4_·7H_2_O, 40.5 µM citric acid and, 0.39 µM Na_2_MoO_4_·2H_2_O [38]. 1 M HCl or NaOH was added to adjust pH to 5.5 and the nutrient solution was replaced every 2 d.

### Nitrogen treatments

For the analysis of gene expression in response to nitrate, rice seedlings were grown in liquid culture for 14 d with 1.44 mM NH_4_NO_3_ as the N source. The solution was changed every 2 d. After 10 d, the medium was changed to 2.88 mM KNO_3_ as the sole N source for 4 d. Then, the seedlings were deprived of N for 3 d before being re-supplied with KNO_3_ or KCl to a final concentration of 2.88 mM. The control plants were grown in continuous 2.88 mM KNO_3_ as the sole nitrogen source. The plants from different treatments were harvested at 4 h, 6 h and 8 h after re-supply separately. The roots were frozen in liquid N_2_ and stored at −80°C for later analysis.

For analyses of gene expression in response to different N sources, the plants were grown in complete nutrient solution with 2.88 mM KNO_3_ as the sole nitrogen source for 14 d [39]. Then, the plants were starved for N for 3 d before being transferred to fresh nutrient solution with the same concentration of different N sources. Roots were harvested 3 h later. For the control treatment, the plants were continuously supplied with 2.88 mM KNO_3_ as the only N source. All the nutrient experiments were repeated at least twice with similar results.

### P and S treatments

To initiate different P and S treatments, two-week-old seedlings grown in complete nutrient solution as described above. For the P and S starvation treatments, the complete nutrient solution was replaced with nutrient solution lacking P or S for 3 d with PO_4_^−^ or SO_4_^2−^ being replaced by chloride. For the control plants, the seedlings grown in continuous complete nutrient solution were transferred to fresh complete nutrient solution at the same time. For re-supply, the appropriate nutrient, 0.32 mM PO_4_^−^ or 2.1 mM SO_4_^2−^ was added in the light period for 3 h before the roots were harvested and frozen in liquid N_2_ and stored at −80°C for gene expression analyses [38].

### RNA extraction and qPCR

Primers for *OsMADS23, OsMADS25, OsMADS27, OsMADS57* and *OsMADS61* for qPCR (see Table 1) were designed using Primer Premier 5. Total RNA was extracted using the RNAiso Plus reagent (Takara) according to the manufacturer’s instructions. The first-strand cDNA was synthesized using PrimeScript RT reagent Kit with gDNA Eraser (Takara). The qPCR was performed in 96-well plates using SYBR Premix Ex Taq II (Takara) according to the manufacturer’s instructions. The qPCR was conducted in the following cycling conditions: 95°C for 30 s, followed by 40 cycles of 95°C for 10 s and 60°C for 30 s. Melt curve analysis was used to confirm the absence of non-specific amplification products. Relative expression levels were calculated by subtracting the threshold cycle (Ct) values for *OsActin* (Os03g0718100) from those of the target gene (to give △Ct) and then calculating 2–△Ct as we described before [40]–[42]. RT-PCR experiments were performed with three biological replicates with the representative being shown.

**Table 1:** List of primers used for Real-time PCR.

Gene	Forward primer (5′–3′)	Reverse primer (5′–3′)
*OsMADS23*	TCTTCTCCAGCACCAGCCGTCT	TGCTGCCTCCTGTTGCCAAAGC
*OsMADS25*	CCAGCTCAAGCATGAAATCAA	AAAGTTGCCTGTTGTTGTGGTGT
*OsMADS27*	GAAGCGGAGGAACGGGATCTTCAA	TGCCATACCGATCTATAACTGACT
*OsMADS57*	ACGAGCAGGCAGGTGACGTT	ACTCATAGAGCCTGCCGGTGCT
*OsMADS61*	GGGAGGGGCAAGATAGTGAT	TGGTGCTGGCATACTCGTAG
OsActin	CTTCATAGGAATGGAAGCTGCGGGT	CGACCACCTTGATCTTCATGCTGCT

### Statistics

The results were analyzed for variance by IBM SPSS Statistics 20. Student’s t-test was calculated at the probability at either at 1% (P<0.01 with significant level **) or 5% (P<0.05 with significant level *) as described before [43], [44].

## Results

### Five *ANR1*-like genes have different expression patterns in response to nitrate

**Figure 1:**
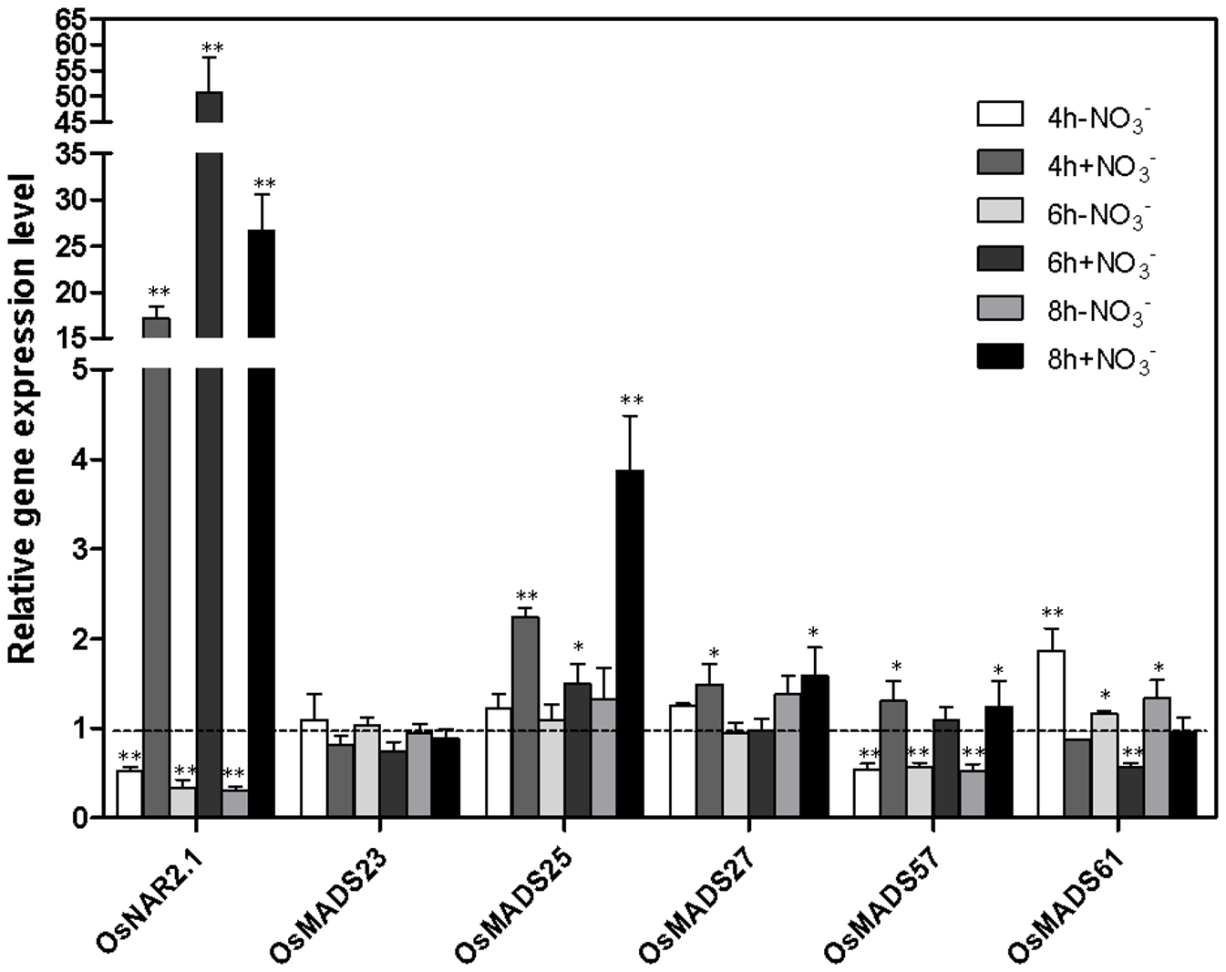
Effect of N deprivation and nitrate resupply on the expression of five *ANR1* related genes in rice roots. Ten-day old rice seedlings grown in complete nutrient solutions were transferred to modified nutrient solutions during which 2.88 mM KNO_3_ was the sole nitrogen source for 4 days. Transcript abundance was assayed by qPCR and was expressed relative to the abundance in roots of plants of the same age grown under continuous N (CK). The *OsNAR2.1* gene, a known nitrate-regulated gene, was included for comparison. Treatments: CK: continuous KNO_3_; −N4h: starved of N for 3 d and resupplied with KCl for 4 h; +N4h: resupplied with KNO_3_ for 4 h; −N6h: starved of N for 3 d and resupplied with KCl for 6 h; +N6h: resupplied with KNO_3_ for 6 h. The mRNA of *OsActin* was used as the reference. A Student’s t-test was calculated at the probability of either 5% (*, p<0.05) or (**, P<0.01).

The *Arabidopsis ANR1* gene was identified as a key gene controlling lateral root growth through NO_3_^−^ signaling [26]. To understand whether these five *ANR1* homologous genes in rice could play similar roles to *ANR1* in *Arabidopsis*, we begin by investigating their expression patterns and levels in response to nitrate. Rice seedlings were grown in complete nutrient solution for 14 d with 2.88 mM KNO_3_ as the sole N source They were then deprived of N for 3 d before being re-supplied with 2.88 mM KNO_3_ (or 2.88 mM KCl as control). The rice nitrate transporter gene *OsNAR2.1*, which is known to be nitrate-inducible [45], was used as a positive control. As shown in Figure 1, the expression of *OsNAR2.1* was very strongly upregulated by nitrate re-supply as expected. Each of the five *ANR1*-related genes was found to have a different expression pattern in response to nitrate starvation and re-supply (Figure 1). Similar to *ANR1* gene in *Arabidopsis*, the expression of *OsMADS61* was significantly induced by nitrate starvation at 4 h, 6 h and 8 h and significantly suppressed by nitrate re-supply at 6 h in comparison to the seedlings continuously supplied with nitrate. In contrast, the expression of *OsMADS25* was not significantly affected by nitrate starvation but was induced by nitrate re-supply at 4 h, 6 h and 8 h in comparison to the continuous nitrate treatment. Similarly, the expression of *OsMADS27* was not significantly affected by nitrate starvation but was induced by nitrate re-supply at 4 h and 8 h in comparison to the continuous nitrate treatment. The expression of *OsMADS57* was downregulated by nitrate starvation at 4 h, 6 h and 8 h and was upregulated by nitrate re-supply at 4 h in comparison to the continuous nitrate treatment (Figure 1). *OsMADS23* was the only gene to show no significant response to either nitrate starvation or nitrate re-supply (Figure 1).

### Five *ANR1*-like genes respond differently to different N sources

**Figure 2:**
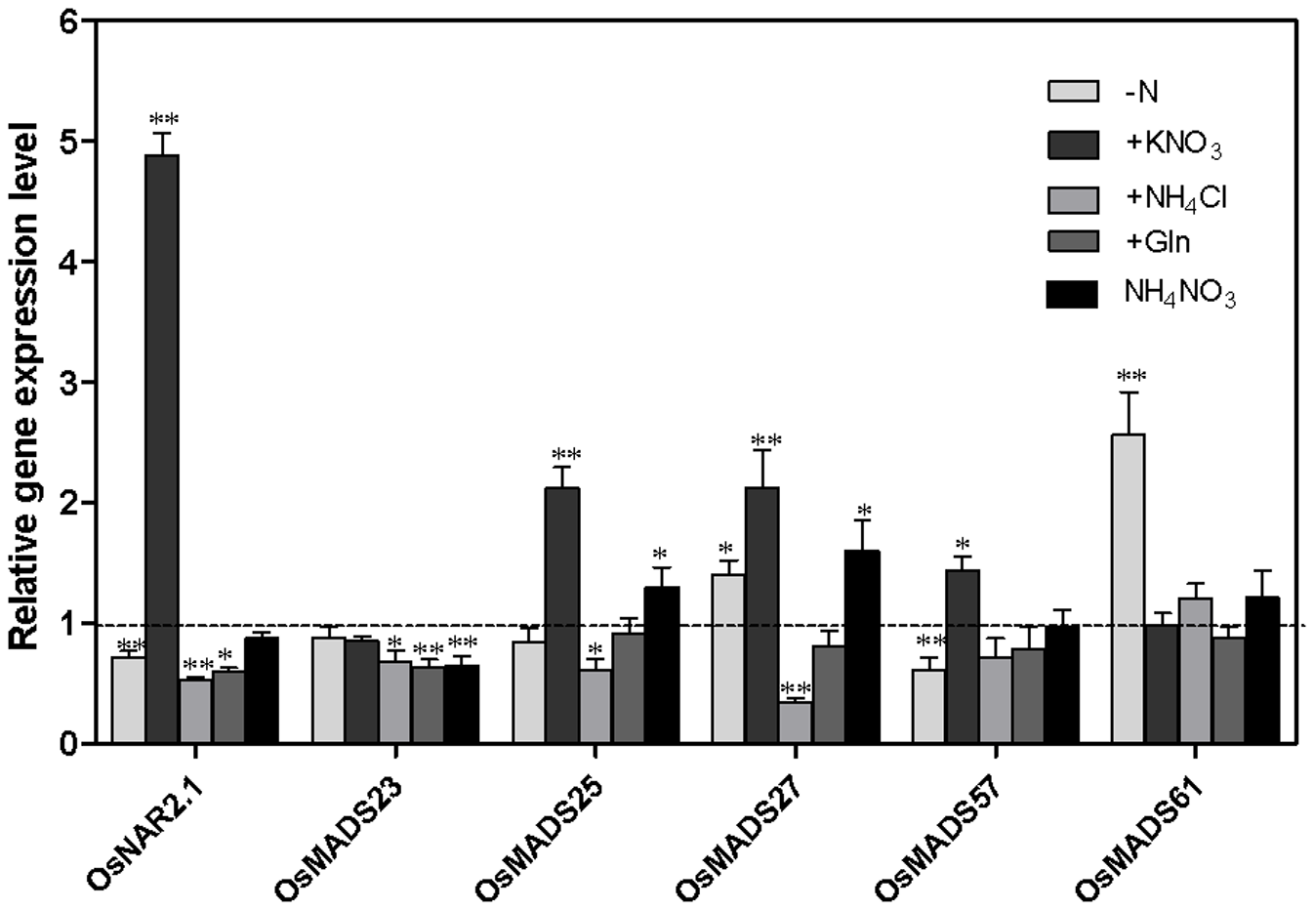
Effect of different N sources on the expression of five *ANR1*-related genes in rice roots. Rice seedlings were grown in liquid culture for 14 days with 2.88 mM KNO_3_ as the sole nitrogen source and then N starved for 3 d. CK, continuous N; −N, starved for 3 d; +KNO_3_, resupplied with 2.88 mM nitrate; +NH_4_Cl resupplied with 2.88 mM NH_4_^+^; and +Gln, resupplied with 2.88 mM glutamine; +NH_4_NO_3_, resupplied with 1.44 mM NH_4_NO_3_. The value of related genes were normalized to its CK control respectively. The mRNA of *OsActin* was used as the reference. Error bars represent _SE. LSD_ values were calculated at the probability of either 5% (*, p<0.05) or (**, P<0.01).

To investigate whether these five *ANR1* homologous genes in rice were also regulated by the other N sources, we further investigated their expression patterns in response to different N sources. The plants were grown in the complete nutrient solution for 10 d before changing media with 2.88 mM KNO_3_ as the sole nitrogen source for 4 days. Then, the plants were starved for N nutrient for 3 d followed by re-supply with the same concentrations of different N sources. As shown in Figure 2, the expression of rice transporter *OsNAR2.1* was down–regulated in the roots of N-starved rice plants and then rapidly upregulated when supplied with 2.88 mM KNO_3_ for 3 h which was consistent with a previous report [39]. *OsMADS61* and *OsMADS57* were the only genes that were affected by nitrate not by any other N sources, whereas *OsMADS23* was the only gene regulated by various different N sources but not by nitrate. In contrast, *OsMADS25* and *OsMADS27* were upregulated by both nitrate and ammonium nitrate but downregulated by ammonium chloride.

### Effect of P and S deprivation and re-supply on the expressions of five *ANR1*-like genes in rice roots

**Figure 3:**
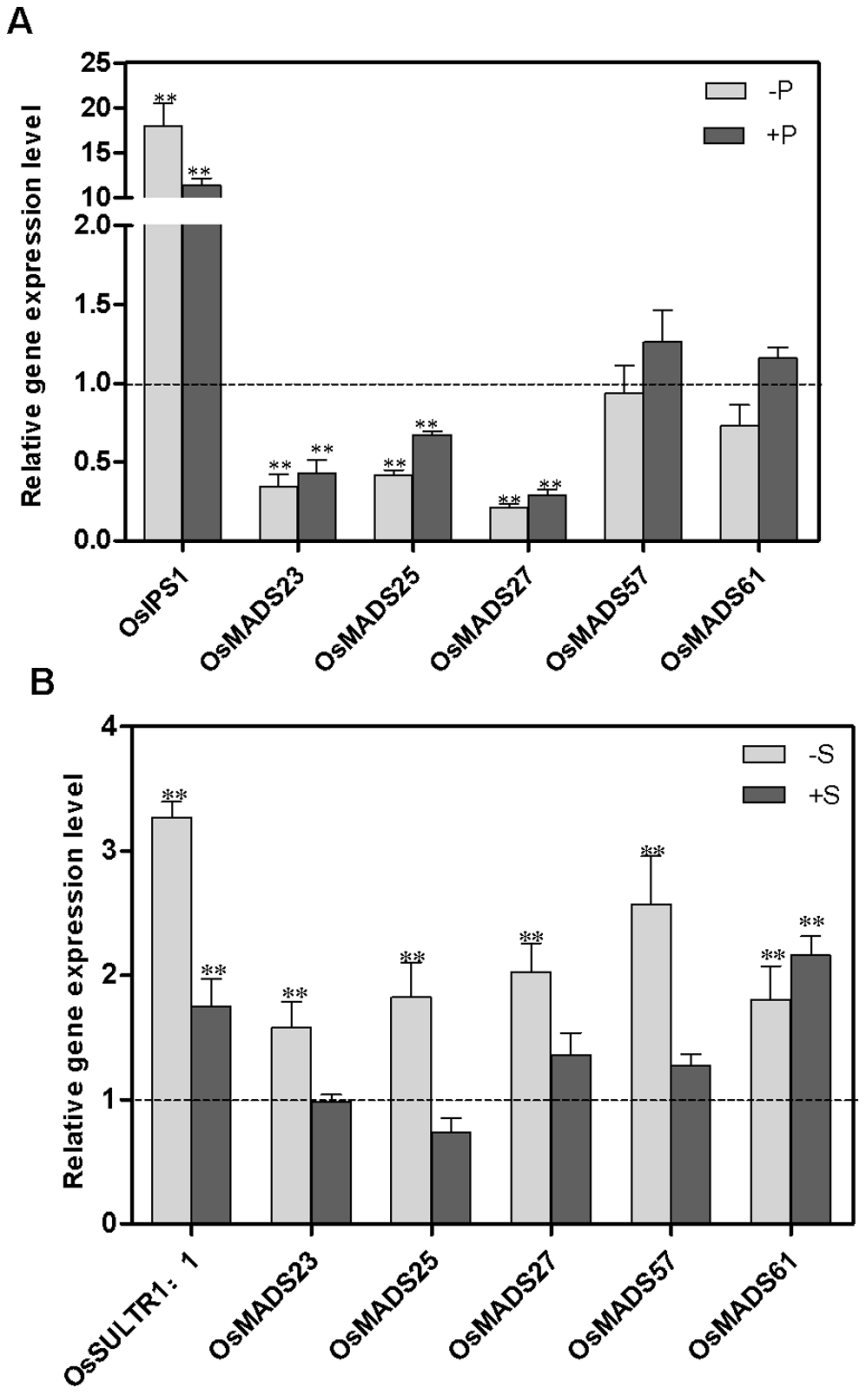
Effect of deprivation and re-supply of phosphate (P) and sulfate (S) on the expression of five *ANR1*-related genes in rice. Two-week old rice seedlings grown hydroponically in complete nutrient solution were deprived of P or S or were maintained on complete nutrient supply for 3 d. In the light period on the day of the experiment, one set of the P-starved and S-starved plants were re-supplied with 0.32 mM H_2_PO_4_^−^ and 2.1 mM SO_4_^2−^ respectively. Roots were harvested 3 h later from controls: continuous nutrient supply (CK); P-deprived (−P); P-resupply (+P); S-deprived (−S); S-resupply (+S). Total RNA was extracted from roots and qPCR reactions were performed in triplicate for each RNA sample. The mRNA of *OsActin* was used as the reference. The value of related genes were normalized to its CK control respectively. A Student’s t-test was calculated at the probability of either 5% (*, p<0.05) or (**, P<0.01).

To analyze whether *ANR1*-like homologous genes are involved in the regulation of gene expression in response to other nutritional stresses, we investigated the effect of P and S deprivation and re-supplementation. As shown in Figure 3, in contrast to the phosphate transporter *OsIPS1*, *OsMADS23, OsMADS25* and *OsMADS27* were all downregulated by P starvation and P re-supply whereas *OsMADS57* and *OsMADS61* were not significantly affected. For the S treatment, *OsMADS23, OsMADS25, OsMADS27* and *OsMADS57* were all upregulated by S starvation but not by S re-supply whereas *OsMADS61* was upregulated by both S starvation and re-supply in comparison to the continuous S nutrient treatment.

### Effect of N-deprivation and re-supply on the expressions of *OsNRT2.1*, *OsNAR2.1*, *OsIPS1* and *OsSULTR1;1* in rice roots

**Figure 4:**
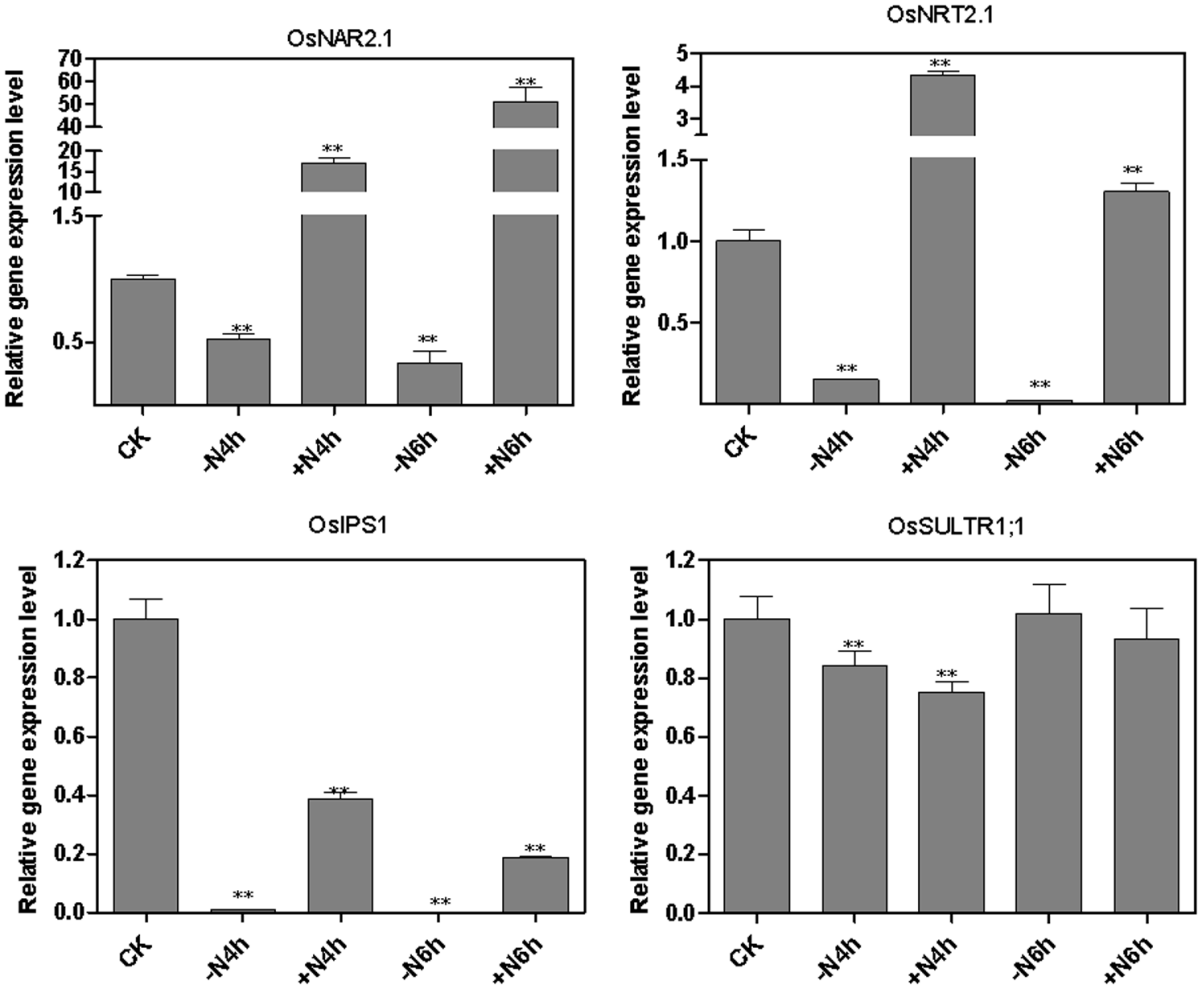
Effect of N-deprivation and re-supply on expression of *OsNRT2.1*, *OsNAR2.1*, *OsIPS1* and *OsSULTR1;1* in rice roots. Rice seedlings were grown hydroponically in a growth cabinet. Nitrogen treatments were as described in Fig. 1. CK: continuous KNO_3_; −N4h: starved of N for 3 d and resupplied with KCl for 4 h; +N4h: resupplied with KNO_3_ for 4 h; −N6h: starved of N for 3 d and resupplied with KCl for 6 h; +N6h: resupplied with KNO_3_ for 6 h. Total RNA was extracted from roots and qPCR reactions were performed in triplicate for each RNA sample. The mRNA of *OsActin* was used as the reference. A Student’s t-test was calculated at the probability of either 5% (*, p<0.05) or (**, P<0.01).

We have investigated whether there is crosstalk between the N, P and S regulatory pathways in the regulation of expression of the the *OsNRT2.1*, *OsNAR2.1*, *OsIPS1* and *OsSULTR1;1* genes. We first investigated whether the expression of phosphate transporter *OsIPS1* and sulphate transporter *OsSULTR1;1* was regulated in response in nitrate starvation and nitrate re-supply. The *OsNRT2.1* and *OsNAR2.1* genes, which encode components of the high affinity nitrate transport system (HATS) in rice, were used as the positive controls *OsNRT2.1* and *OsNAR2.1*, were previously shown to be upregulated by nitrate and suppressed by NH_4_^+^. As expected, *OsNRT2.1* and *OsNAR2.1* were downregulated by nitrate starvation and rapidly upregulated by nitrate re-supply (Figure 4), confirming the results of previous studies [45], [46]. However, the expression of phosphate transporter *OsIPS1* was significantly downregulated by both nitrate starvation and nitrate re-supply at both 4 h and 6 h time points, whereas *OsSULTR1;1* was only significantly downregulated by both nitrate starvation and re-supply at 4 h and not at 6 h.

### Effect of P, S deprivation and re-supply on the expression of *OsNRT2.1*, *OsNAR2.1*, *OsIPS1* and *OsSULTR1;1* in rice roots

**Figure 5:**
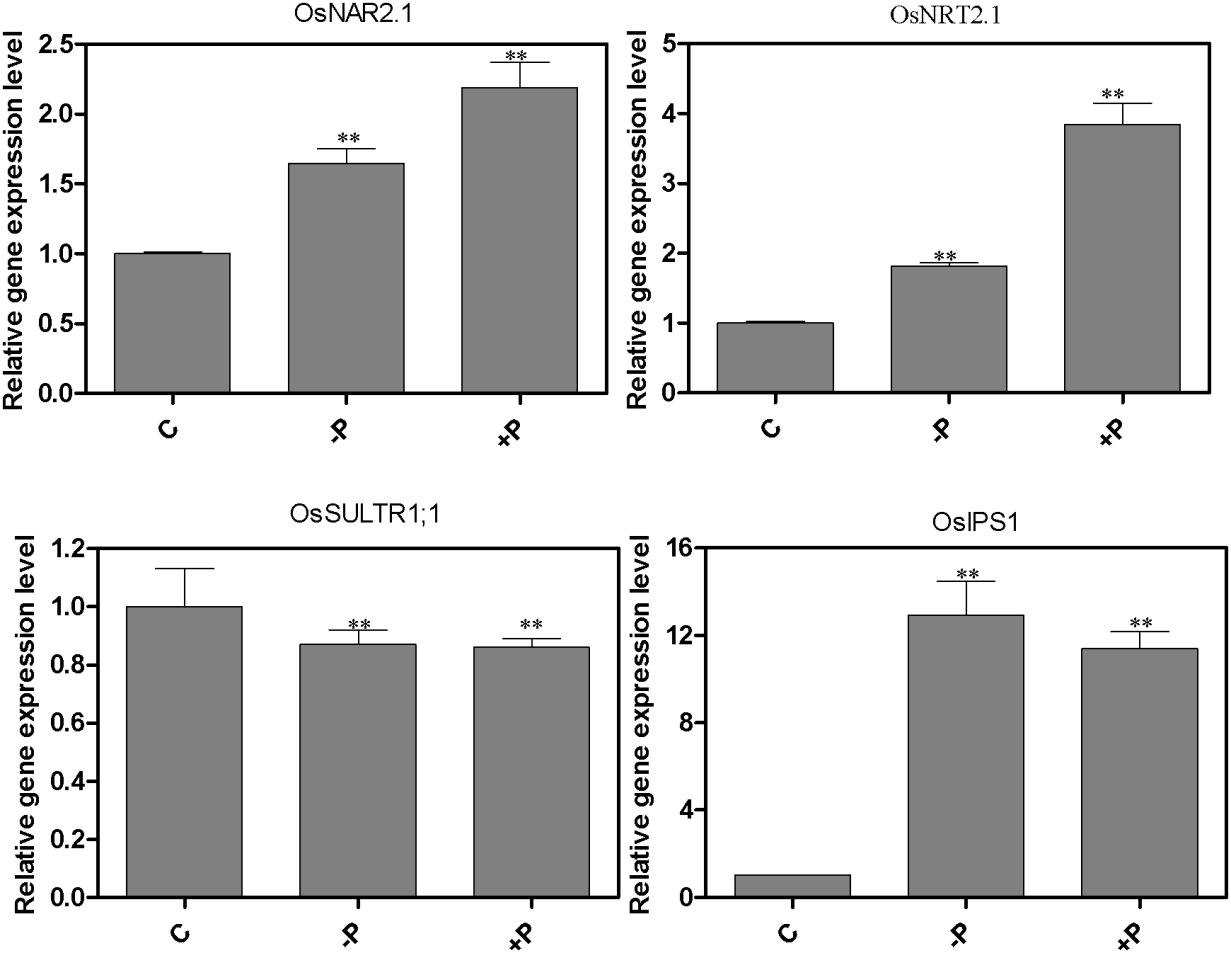
Effect of P-deprivation and re-supply on expression of *OsNRT2.1*, *OsNAR2.1*, *OsIPS1* and *OsSULTR1;1* in rice roots. Rice seedlings were grown hydroponically in a growth cabinet. Phosphorous treatments were as described in Fig. 3. C: continuous complete nutrient supply; −P: starved of P; +P: resupplied with H_2_PO_4_^−^ for 3 h. Total RNA was extracted from roots and qPCR reactions were performed in triplicate for each RNA sample. The mRNA of *OsActin* was used as the reference. A Student’s t-test was calculated at the probability of either 5% (*, p<0.05) or (**, P<0.01).

**Figure 6:**
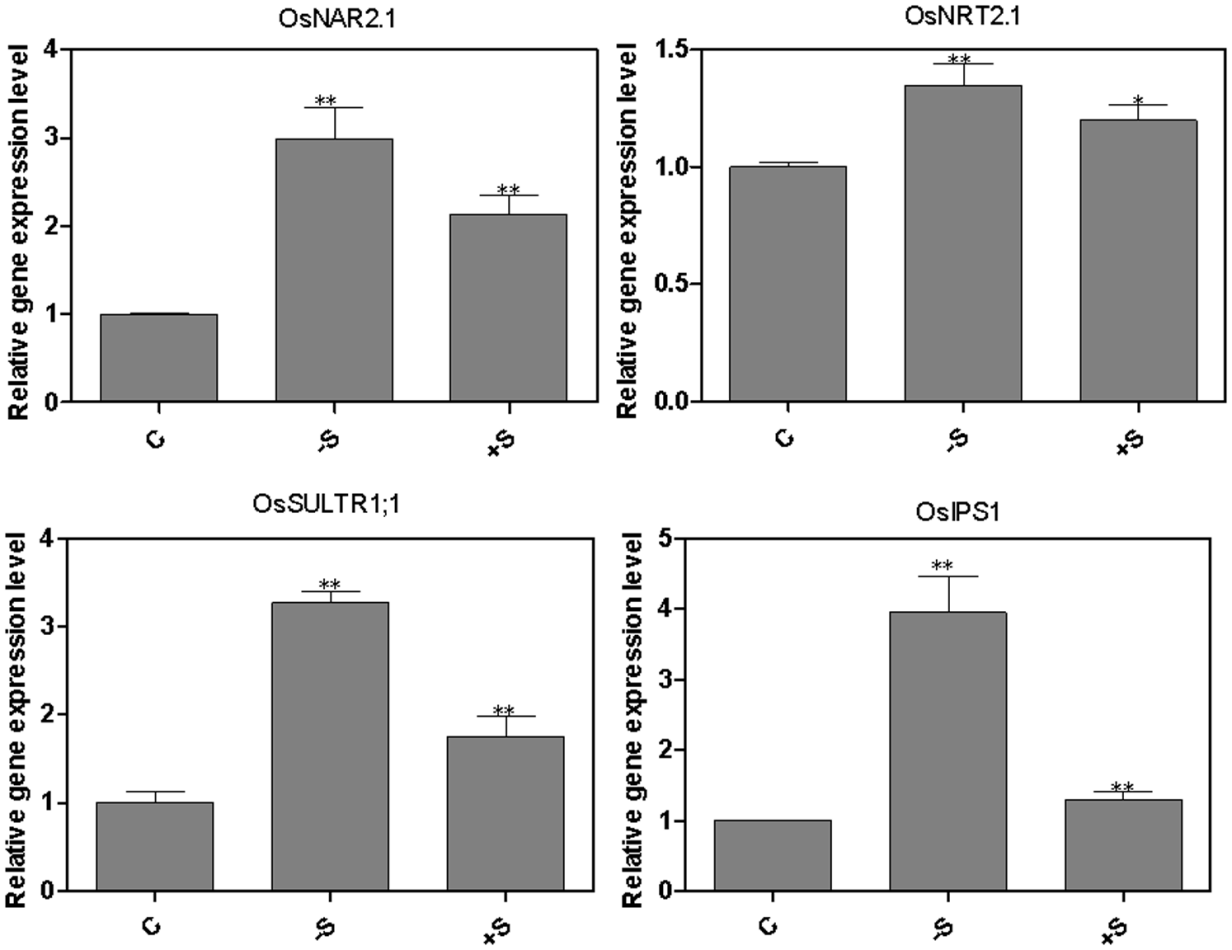
Effect of S-deprivation and re-supply on expression of *OsNRT2.1*, *OsNAR2.1*, *OsIPS1* and *OsSULTR1;1* in rice roots. Rice seedlings were grown hydroponically in a growth cabinet. Sulfur treatments were as described in Fig. 3. C: continuous complete nutrient supply; −S: starved of S; +S: resupplied with SO_4_^2−^ for 3 h. Total RNA was extracted from roots and qPCR reactions were performed in triplicate for each RNA sample. The mRNA of *OsActin* was used as the reference. A Student’s t-test was calculated at the probability of either 5% (*, p<0.05) or (**, P<0.01).

In this experiment two-week old rice seedlings grown in complete nutrient solution were deprived of phosphate or sulfate for 3 d and re-supplied with phosphate or sulfate for 3 h. *OsIPS1* was used as P control gene and *OsSULTR1;1* was used as S control gene. As shown in Figure 5, the expression of *OsIPS1* was notably upregulated by P deprivation and downregulated by P re-supply, which was consistent with previous study [14]. Surprisingly, the gene expression patterns of *OsNRT2.1* and *OsNAR2.1* is very similar to the expression pattern of *OsIPS1*, which were upregulated by both starvation and P re-supply in comparison to the continuous P treatment (Figure 5). However, the expression of *OsSULTR1;1* was significantly downregulated by P starvation and P re-supply in comparison to the continuously P supply treatment. For the S fluctuation treatment, as shown in Figure 6, the mRNA level of *OsSULTR1;1* was significantly increased by sulfate starvation, which was consistent with previous study [47]. The expression patterns of *OsNRT2.1*, *OsNAR2.1* and *OsIPS1* were the same as *OsSULTR1;1*, being upregulated by both S starvation and S re-supply in comparison to the continuous S treatment (Figure 6).

## Discussion

### Five *ANR1*-like genes have different expression patterns in response to nitrogen

It was previously demonstrated that expression of *ANR1* in roots of hydroponically grown *Arabidopsis* plants was induced by N deprivation and rapidly downregulated by N re-supply [29]. This pattern of N responsiveness differs from the NO_3_^−^ inducibility of *ANR1* as previously obtained in *Arabidopsis* root cultures [26]. In the present study we have shown that the five *ANR1-*related genes in rice have diverse expression patterns in response to N starvation and N re-supply. The expression of *OsMADS61* was only significantly induced by N starvation and significantly down regulated by N re-supply, which is very similar to the expression pattern of *ANR1* in *Arabidopsis.* In contrast, the expression of *OsMADS57* was significantly downregulated by N starvation and significantly upregulated by nitrate re-supply. Furthermore, *OsMADS25* and *OsMADS27* were only significantly upregulated by nitrate re-supply not by N starvation. These results were partly consistent with the results from [48], in which the rice seedlings were grown in half-strength liquid Murashige and Skoog medium without N and re-supplied with 3 mM KNO_3_. They found that *OsMADS23* and *OsMADS61* were not significantly affected by N fluctuation and that *OsMADS25, OsMADS27, OsMADS57* were all significantly upregulated by nitrate re-supply [48]. They therefore suggested that the *AGL17*-like related genes in rice had specific functions differing from those of their *A. thaliana* homologs. However, our finding found that the expression pattern of *OsMADS61* is very similar to that of *ANR1* pattern in *Arabidopsis*.

It has recently been shown that microRNA444 (miR444), which targets three OsMADS-box genes (*OsMADS23, OsMADS27, OsMADS57)* in rice, has multiple roles in the NO_3_^–^ signaling pathway [49]. In this study, *OsMADS23, OsMADS27* and *OsMADS57* were downregulated under conditions of N-deprivation and were unaffected following 0.5, 1, or 2 h of 5 mM KNO_3_ supplementation [49]. The different sampling times may account for the partial differences between their results and ours.

The phenotype of transgenic lines overexpressing miR444 provided evidence that *OsMADS23*, *OsMADS27* and *OsMADS57* could have a role in regulating the lateral response to localised nitrate in rice [49], as *ANR1* does in *Arabidopsis* [26]. In addition, there was evidence that this group of genes is involved in controlling the root architecture in response to P starvation [49]. However, it has been noted that miR444 has additional target genes (non-MADS box) in rice and there could be other unknown genes involved in the root developmental responses to the nutrient supply [50].

### The five *ANR1*-like homologous gene in rice are regulated by P and S fluctuations

Previous results from *Arabidopsis* had demonstrated that expression of *ANR1* was not regulated by fluctuations in P and S supplies and that and *SUPPRESSOR OF OVEREXPRESSION OF CONSTANS 1* (*SOC1*) was the only type-II MADS-box gene that responded to phosphate and sulfate deprivation and re-supplementation [29], [41]. A later study found that *AGL12*, *AGL18* and *AGL19* were downregulated by P and S re-supply [41]. In this study, the *OsMADS23*, *OsMADS25* and *OsMADS27* genes were downregulated by both P starvation and P re-supply whereas expression of *OsMADS57* and *OsMADS61* was not significantly regulated by P fluctuations, which was consistent with what was observed for *ANR1* in *Arabidopsis* [29]. These results were partly consistent with the results from [49], in which phosphate starvation reduced the mRNA levels of two miR444 targets (*OsMADS27* and *OsMADS57*). In our result, the expression level of *OsMADS23* and *OsMADS27* were downregulated by P starvation, however in [49], the mRNA abundance of *OsMADS27* and *OsMADS57* were downregulated by phosphate starvation. We used Yoshida [38] rice nutrient solution and Yan chose 1/2 MS culture solution [49] and the time course of phosphate-deprivation was different, which may account for the different gene expression patterns. However, for the S treatment, the expression of all five *ANR1*-related genes in rice was modulated by S starvation, in contrast to what was seen with *ANR1* in Arabidopsis [29], suggesting that these rice genes have specific functions differing from their *Arabidopsis* homologs. Furthermore, the result of the expression of *OsMADS25* regulated by phosphate deprivation was consistent with previous report by [51]. Like *SOC1* in *Arabidopsis*, rice *OsMADS25*, *OsMADS27* and *OsMADS57* in roots were responsive to nitrate, phosphate and sulfate fluctuations, which suggest that these three genes may be involved in a general stress response pathway to these three macronutrients.

### Crosstalk among *OsNRT2.1*, *OsNAR2.1*, *OsIPS1* and *OsSULTR1;1*

As already discussed, depriving seedlings of one mineral nutrient may lead to the disruption of the metabolism of other nutrients [29], [52], [53]. For example, molybdenum deficiency had positive impacts on genes involved in nitrate and sulfate assimilation and phosphate transport [54]. Our results show that *OsNRT2.1* and *OsNAR2.1* were regulated by both P and S starvation and re-supply, which are partly consistent with previous research in which the gene expression level of *AtNRT2.1* in *Arabidopsis* was found to be upregulated by P and S re-supply [44]. Our results also indicated that *OsIPS1* was sensitive to N and S fluctuation and that the expression of *OsSULTR1;1* was downregulated by phosphate re-supply, indicating that there is crosstalk between the signaling pathways regulating expression of nitrate, phosphate and sulfate transporters. This is similar to the finding that some nitrate-inducible genes are involved in sulfate metabolism [51], [55]. These results suggest that there is a complex regulatory network among these three macronutrients and their transporters. Further work using mutants or other genomic approach needs to be done to identify the mechanisms of the crosstalk among nitrate, phosphate and sulfate transporters.
